# Effects of mRNA secondary structure on the expression of HEV ORF2 proteins in *Escherichia coli*

**DOI:** 10.1186/s12934-017-0812-8

**Published:** 2017-11-14

**Authors:** Nouredine Behloul, Wenjuan Wei, Sarra Baha, Zhenzhen Liu, Jiyue Wen, Jihong Meng

**Affiliations:** 1 Department of Microbiology and Immunology, School of Medicine, Southeast University, 87 DingJiaQiao Road, Nanjing, 210009 Jiangsu China; 2 Department of Pharmacology, Anhui Medical University, Hefei, 230032 China

**Keywords:** Recombinant protein expression, *Escherichia coli*, RNA secondary structure, Hepatitis E virus

## Abstract

**Background:**

Viral protein expression in *Escherichia coli* (*E. coli*) is a powerful tool for structural/functional studies as well as for vaccine and diagnostics development. However, numerous factors such as codon bias, mRNA secondary structure and nucleotides distribution, have been indentified to hamper this heterologous expression.

**Results:**

In this study, we combined computational and biochemical methods to analyze the influence of these factors on the expression of different segments of hepatitis E virus (HEV) ORF 2 protein and hepatitis B virus surface antigen (HBsAg). Three out of five HEV antigens were expressed while all three HBsAg fragments were not. The computational analysis revealed a significant difference in nucleotide distribution between expressed and non-expressed genes; and all these non-expressing constructs shared similar stable 5′-end mRNA secondary structures that affected the accessibility of both Shine-Dalgarno (SD) sequence and start codon AUG. By modifying the 5′-end of HEV and HBV non-expressed genes, there was a significant increase in the total free energy of the mRNA secondary structures that permitted the exposure of the SD sequence and the start codon, which in turn, led to the successful expression of these genes in *E. coli*.

**Conclusions:**

This study demonstrates that the mRNA secondary structure near the start codon is the key limiting factor for an efficient expression of HEV ORF2 proteins in *E. coli.* It describes also a simple and effective strategy for the production of viral proteins of different lengths for immunogenicity/antigenicity comparative studies during vaccine and diagnostics development.

**Electronic supplementary material:**

The online version of this article (10.1186/s12934-017-0812-8) contains supplementary material, which is available to authorized users.

## Background

Expression of recombinant proteins is a powerful tool for structural and functional studies of numerous gene products and for the production of poly and monoclonal antibodies as well as the production of various pharmaceuticals such as vaccines. Among the available expression systems, the prokaryotic host cells such as *E. coli* are usually preferred due to the easy culturing procedures and to the low-cost production of high protein yields. This makes the prokaryotic systems economically attractive for large scale and cost effective production of recombinant proteins [1–3].

However, high-level expression of heterologous proteins in *E. coli* is still a challenge that mainly depends upon each individual target gene. Although the influence of several factors has been reported, uncertainty remains concerning the importance and the implication extent of each individual factor: Several studies have focused on the relationship between the codon usage and the translation efficiency in *E. coli* and reported that a set of rare codons can disrupt translation [4–8]. The mRNA secondary structure and stability have been also identified as the major factors that influence protein expression in *E. coli* [9, 10]. However, the ribosome-profiling studies revealed that the net translation-elongation rate is generally constant independently of codon usage [11] because of a trade-off between the beneficial/detrimental effects of tRNA abundance and local mRNA secondary structure as reported by Gorochowski et al. [12].

Hepatitis E virus (HEV) and hepatitis B virus (HBV) are the causative agents of hepatitis E and B respectively, two of the major health concerns worldwide [13–17].

Hepatitis B virus is a DNA virus of the Hepadnaviridae family. It is a small, enveloped virus with a circular and partially double-stranded DNA genome of 3.2 kb that replicates exclusively in the liver. The *env* gene of HBV codes for three related proteins: (1) the HBV envelope S protein (HBsAg) which is the constituent of the HBV envelope (226 amino acids), (2) The 55 amino acids (aa) protein encoded by the pre-S2 portion of the pre-S region and (3) a large protein encoded by the whole ORF (pre-S1, pre-S2 and S, 389 aa). Among these proteins, HBsAg has the highest density of neutralizing epitopes [18]. Therefore, the expression of HBsAg was very valuable given its potential use as a vaccine candidate and as a diagnostic antigen. HBsAg was expressed and purified in different eukaryotic and prokaryotic systems with better results in the former. Since the 70 s till the present day, numerous attempts have been made to produce the HBsAg in *E. coli* but the expression levels were too low to be economically viable [19–24].

Hepatitis E virus is a non-enveloped virus of the Hepeviridae family, with a positive-sense single-stranded RNA genome of 7.2 kb that contains three open reading frames (ORFs) [25]. Of these three ORFs, the ORF2 encodes the viral capsid protein of 660 aa which is composed of three domains: the S domain (aa 129–319), the M domain (aa 320–455), and the P domain (aa 456–606) [26]. This latter is responsible for the virus interaction with the target cells and contains the most important neutralization epitopes [26–28]. Therefore, the P domain has been extensively studied for the expression of efficient antigens for both diagnostic and vaccine development purposes.

Several fragments of the ORF2-encoded proteins have been successfully over-expressed in *E. coli:* p166 (aa 452–617), p179 (aa 439–617), p239 (aa 368–606), E2s (aa 454–606), aa 112–607 and aa 458–607 fragments [29–35]. All these fragments have been reported to mimic the antigenicity and immunogenicity of HEV which indicates that the main antigenic region is located within the aa 458–606 portion of the ORF2 protein. However, the reasons for choosing a specific length of the fragment were not reported or unclear. The restriction sites to be used for the insertion of the coding DNA into a vector could affect the choice of the sequence start. More importantly, according to our own experience, several fragments are usually designed in such experiments and then the expressed ones are further investigated while the non-expressed ones are not worth reporting, especially when the expressed proteins are fully functional.

Therefore, in this study, we investigated the effects of codon usage and mRNA secondary structure at the 5′-end on the expressivity of different fragments of HEV ORF2 and HBsAg proteins. Our results revealed that the formation of stable mRNA secondary structure near the start codon disrupted the expression of HEV and HBV proteins and the introduction of mutations that destabilize this stable secondary structure improved the expression of the target proteins.

## Methods

### Reagents

Restriction enzymes, T4 Ligase, Expand High Fidelity PCR System and kanamycin were purchased from Roche Diagnostic (Germany); *E. coli* BL-21(DE3) and pET28a, vector were purchased from Novagen (Germany); Miniprep kit for DNA and plasmid recovery and purification were purchased from Qiagen (Germany). All other chemicals were of analytical grade and purchased from Sigma-Aldrich (Germany).

Recombinant plasmid pET28a(+)/549, containing the HEV-ORF2-encoding DNA of the genotype 4 HEV strain H4-NJ703; and the recombinant plasmid pPICZα-HBs containing the HBV surface antigen-encoding DNA sequence (Accession Number: FJ743736) were previously constructed in our laboratory.

### Plasmid construction

For the following experiments: All PCRs were performed using the Expand High Fidelity PCR System and then PCR products were purified with the Miniprep DNA recovery kit; all digestions with restriction enzymes were carried out at 37 °C for 4 h; all ligations were performed using T4 DNA ligase at 16 °C overnight, and then used to transform *E. coli* competent BL21 cells; all recombinant plasmids were recovered from transformants by using the Plasmid Miniprep kit and the presence of inserts confirmed by PCR, restriction enzymes digestion and DNA sequencing.

The genes encoding the HEV ORF2 recombinant proteins p146 (aa 457–602), E2s (aa 455–602) p216 (aa 422–637), p222 (aa 439–660), p231 (aa 430–660) were amplified from the pET28a/549 recombinant plasmid; the genes encoding the HBV antigens HBsAg-1 (aa 1–226), HBsAg-2 (aa 87–226) and HBsAg-3 (aa 1–205) were amplified from the pPICZα-HBs recombinant plasmid. All of the PCR fragments of the selected genes were modified to contain 5′-*Nco*I and 3′-*Xho*I restriction sites using the primers shown in Table 1. Then, they were digested with *Nco*I and *Xho*I restriction enzymes and cloned downstream of the T7 promoter in the *E. coli* expression vector pET28a(+). All the cloned genes were fused to the pET28a(+) His-tag downstream the *Xho*I restriction site, which facilitates the detection and purification of the recombinant protein on one hand and keeps the same 3′ end for all the genes on the other hand. Finally, the expression constructs were verified by sequence analysis to ensure that all of the genes were inserted correctly.

**Table 1:** List of the forward (F) and reverse (R) primers used for the amplification of the different DNA coding sequences

Primers	Sequences
146-NCOI-F	TTT **CCATGG** CTCCTTCTCGCCCTT
Mutated 146-NCOI-F	CCC **CCATGG** ATGATTCTCGCCCTT
146-XHOI-R	TTT **CTCGAG** TGCGAGGACACCGAC
*Nco*I-E2s-NCOI-F	TTT **CCATGG** TTTCCCCTGCTCCTTCTCG
Mutated *Nco*I-E2s-NCOI-F	CCC **CCATGG** TTTCCCCTGATGATTCTC
*Nco*I-E2s-XHOI-R	TTT **CTCGAG** TGCGAGGACACCGAC
216-NCOI-F	CCC **CCATGG** ATAAGGGGATAGCTAT
216-XHOI-R	CCC **CTCGAG** GCCCTGAAGGCCGAGC
222-NCOI-F	GGG **CCATGG** TTATCCAGGACTATGA
222-XHOI-R	CCC **CTCGAG** ATACTCCCGGGTTTTACCCC
231-NCOI-F	CCC **CCATGG** ATATTGATCTTGGTGAGTCC
231-XHOI-R	CCC **CTCGAG** ATACTCCCGGGTTTTACC
HBsAg-1-NCOI-F	CCC **CCATGG** AGAGCACAACATCAGGA
Mutated HBsAg-1-NCOI-F	CCC **CCATGG** AGAGCACAACATCAGATTTCC
HBsAg-1-XHOI-R	CCC **CTCGAG** AATGTATACCCAAAG
HBsAg-2-NCOI-F	CCC **CCATGG** CTCTGCTGCTATGCCTCATC
Mutated HBsAg-2-NCOI-F	TTT **CCATGG** CTCTGCTTCTGTGCCTC
HBsAg-2-XHOI-R	CCC **CTCGAG** AATGTATACCCAAAG
HBsAg-3-NCOI-F	CCC **CCATGG** AGAGCACAACATCAGGATTC
HBsAg-3-XHOI-R	CCC **CTCGAG** CAGACTTGGCCCCCAATACC

The restriction sites are indicated in bold

The positions of the mutations introduced into the mutated genes are underlined

### Protein expression and purification

For each protein, BL21 (DE3) cells were transformed with the appropriate plasmid and plated on LB agar with 50 μg/ml kanamycin. A single colony was picked and grown overnight at 37 °C in Luria–Bertani broth (LB) containing 50 μg/ml kanamycin (LB/Kan+) as a starter culture. The overnight culture was diluted 1:100 in 500 ml of LB/Kan+ and grown to an OD_600_ between 0.6 and 0.7. The expression was induced for 3 h by the addition of IPTG to a final concentration of 0.5 mM. To verify the expression of the target proteins, the cells were collected by centrifugation and the pellet was analyzed by 15% sodium dodecyl sulfate–polyacrylamide gel electrophoresis (SDS–PAGE). For large scale purification, cultures were harvested by centrifugation in a Beckman Allegra™ 21R centrifuge at 6000 rpm, 4 °C, 20 min. Cell pellets were stored at − 80 °C prior to lysis and protein purification. All the proteins were C-terminally His-tagged and purified by Ni–NTA affinity chromatography. Briefly, cell pellets were resuspended in a lysis buffer (50 mM NaH_2_PO_4_, pH 8.0, containing 300 mM NaCl and 10 mM imidazole) and lysed with lysozyme, the suspension was clarified by centrifugation and then the supernatant was loaded onto a column containing Ni–NTA superflow affinity resin, equilibrated with 50 mM NaH_2_PO_4_, PH 8.0, containing 300 mM NaCl and 10 mM imidazole buffer. The column was washed with five volumes of equilibration buffer, and the fusion proteins were eluted with the same buffer containing 250 mM imidazole. The fractions containing proteins were analyzed by SDS-PAGE. The relevant fractions were pooled and stored at − 80 °C for further use.

### Western blotting

Ni–NTA purified proteins were run on 15% denaturing gel (SDS-PAGE), along with pre-stained protein markers on adjacent lanes and transferred electrophoretically to nitrocellulose membranes. The membranes were incubated overnight with blocking buffer containing 5% fat-free milk, 0.05% Tween 20 in 0.01 M Tris buffered saline (TBS) and then incubated for 1 h with anti-HEV 4C5 or anti-HBV monoclonal antibody diluted in 1:2000 in blocking buffer. The membranes were rinsed three times with wash buffer (TBS with 0.05% Tween 20) and incubated for 1 h with affinity-purified goat anti-human immunoglobulin G conjugated with horseradish peroxidase diluted 1:2000 in blocking buffer. After three washes, color development was carried out with 3, 3′-diaminobenzidine (DAB) as substrate.

### In silico analysis

For the in silico analysis, besides the proteins targeted in this study we also analyzed the proteins that have been previously expressed, namely the p166 (aa 452–617), p179 (aa 439–617) and *Bam*HI-E2s (E2s inserted into the 5′*Bam*HI–3′*Xho*I) [data not published].

The nucleotide composition, codons usage and the adaptation codon index (CAI) were analyzed by GenScript Rare Codon Analysis Tool (https://www.genscript.com/tools/rare-codon-analysis) and CAIcal server [36]. To investigate the patterns of synonymous codon usage (RSCU) without the influence of amino acid composition among HEV and HBV target genes, the RSCU values of codons were calculated using CAIcal server.

For each gene, the secondary structure of the mRNA (− 43/AUG/+ 43) sequence containing the ribosome-binding site (RBS), translation initiator AUG and the first 14 codons, was predicted and analyzed using the Rtools web server (http://rtools.cbrc.jp/cgi-bin/index.cgi). This server provides a rich set of tools for analyzing a single RNA sequence:

- We used the CentroidFold tool for the structure prediction [37]. Based on a generalized centroid estimator, CentroidFold is one of the most accurate tools for predicting RNA secondary structures.
- Then, we used the Raccess algorithm [38] to compute the accessibility of segment [a, b] = [x, x + l − 1] in the RNA sequence for all the positions x with fixed length l (Acc.len) = 5, 10. The thermodynamic energy that is required to keep range [a, b] being accessible is given by:
  c. access_energy([a, b]) = − RT log(prob([a, b]))
  d. prob([a, b]) = sum_{s in S[a, b] exp(-E(s)/RT)/sum_{s in S0} exp(-E(s)/RT)
  where S0 is all the possible secondary structures of the transcript and S[a, b] is all the secondary structures having range [a, b] as loop region.
- We also analyzed the secondary structures using CapR algorithm [39] which calculates for each RNA base the probabilities to be located within six different categories of RNA structures: stem part, hairpin loop, bulge loop, internal loop, multi-branch loop, and exterior loop. Then the results show a structural profile of an RNA base by a set of six probabilities that the base belongs to each category.
- Finally, we used the Rchange algorithm [40] to compute the entropy and the internal energy changes of the secondary structures for each single-point mutation.

### Modification of the 5′-end of the non-expressed genes

The 5′-end of the non-expressed genes were mutated according to the results obtained from the analysis of single-point mutation effects on the free energy changes of the RNA secondary structure. The secondary structure of the mRNA 5′ end of the mutated genes were analyzed as described above.

For the protein expression, new set of primers were designed to harbor the mutations (Table 1). Then, the fragments were amplified, purified, digested with endonucleases (*Nco*I and *Xho*I) and inserted into the pET28a(+) vector. After sequence analysis to confirm the insertion of the mutations, the constructs were used to transform competent *E. coli* (BL21) cells. The protein expression, purification and Western blot were conducted as described in the previous sections.

## Results

### Expression of the target genes in *E. coli*

**Fig. 1:**
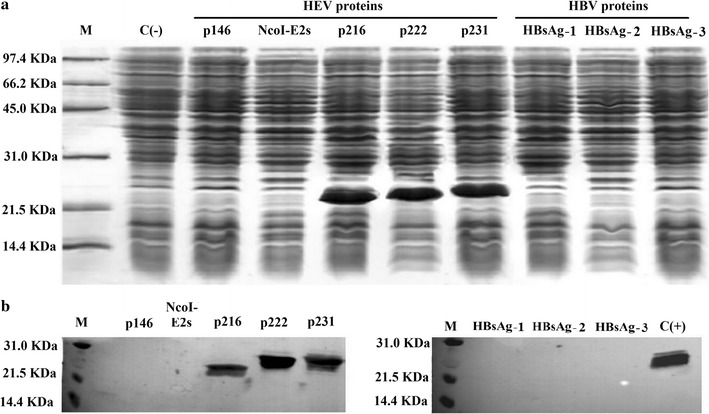
Analysis of protein expression by SDS-PAGE and Western blot. **a** A 15% SDS-PAGE presenting the expression under IPTG induction. It shows the overexpression of p216, p222 and p231 at expected sizes of 24.7, 25.5 and 26.5 kDa respectively; while no extra-bands are visible for p146 (16.7 kDa), *Nco*I-E2s (16.9 kDa), HBsAg-1 (26.5 kDa), HBsAg-2 (17.1 kDa) and HBsAg-3 (23.9 kDa), compared to the control. *M* molecular weight marker, *C(−)* negative control, before IPTG induction. **b** Western blot analysis using anti-HEV 4C5 monoclonal antibody for HEV proteins and anti-HBsAg monoclonal antibody for HBV proteins. *M* molecular weight marker, *C(+)* positive control

The plasmid pET28a(+) was used to express the target genes. All the genes were inserted into the 5′*Nco*I and 3′*Xho*I restriction sites and confirmed with DNA sequencing. Then, the constructs were successfully transformed into BL21(DE3) host cells. For each gene, several colonies of transformed *E. coli* were picked out and subjected to IPTG induction to identify clones capable of expressing the recombinant proteins. As shown in Fig. 1a, three of the HEV ORF2 proteins were over-expressed, namely p216, p222 and p231, while the p146, E2s, HBsAg-1, HBsAg-2 and HBsAg-3 were not. The plasmids were recovered from the clones used for the recombinant protein expression and restriction analysis/DNA sequencing showed that all the genes were correctly inserted into the designated sites without any shifting or mutations.

Further, the optimization of the expression conditions by testing different IPTG concentrations (0.3–3.0 mM) and induction durations (0.5–8 h), revealed that IPTG was effective at all concentrations; the protein accumulation increased up to 3 h and stayed constant thereafter (data not shown). After Ni–NTA resin purification, the activity of the recombinant protein was analyzed by Western blotting as shown in Fig. 1b. The three expressed proteins showed an equally strong reactivity against the anti-HEV monoclonal antibody.

### Nucleotide composition, codon usage and codon adaptation of the target genes

The results are shown in Table 2. In this analysis, the genes were divided into two groups: expressed genes (p166, p179, p216, p222, p231 and *Bam*HI-E2s) and non-expressed genes (p146, *Nco*I-E2s, HBsAg-1, HBsAg-2 and HBsAg-3). The results revealed that there is no significant difference in the overall percentage of the different nucleotides or the G + C percentage between the expressed and non-expressed genes. However, by analyzing the nucleotide composition at the three positions of the codons, we found that there were relatively significant differences: at the first position, the expressed genes contained more guanine (G) and less thymine (T) than the non-expressed genes which led to a significant difference in the G1 + C1 percentage; at the second position of the codons, the expressed genes contained more adenine (A) and less thymine (T) than the non-expressed genes; at the third position, the expressed genes contained more thymine (T) and less adenine (A) than the non-expressed genes. However, the analysis of the amino acid composition of the target proteins; and the relative synonymous codon usage in HEV and HBV target genes (Additional file 1: Tables S1, S2) revealed that the differences in nucleotide composition were related to the difference in the amino acid composition between HEV and HBV target proteins. Concerning the codon usage, there was no difference in the codon adaptation index (CAI) or in the expected CAI between the two groups.

**Table 2:** Nucleotide composition and codon adaptation index of the expressed and non-expressed genes

	Parameters	Expressed genes		Non-expressed genes	
		Average	SD	Average	SD
Nucleotide composition	A	18.68	0.46	18.83	0.53
	C	26.75	0.29	26.82	1.05
	T	32.32	0.96	32.83	1.53
	G	22.25	0.92	21.52	0.52
	G + C	49	0.72	48.34	1.17
	A1^a^	22.63	1.8	23.28	1.24
	C1	23.98	1.03	24.82	2.34
	T1	*21.46*	*1.23*	*29.07*	1.81
	G1	*31.93*	*1.45*	*22.84*	2.13
	G1 + C1	*55.91*	*1.95*	*47.65*	1.44
	A2^b^	*26.79*	*2.12*	*17.07*	3.83
	C2	34.17	2.35	32.13	3.29
	T2	*23.75*	*0.52*	*30.95*	1.1
	G2	15.3	2.7	19.85	2.73
	G2 + C2	49.46	2.11	51.98	1.79
	A3^c^	*6.63*	*0.34*	*16.13*	1.56
	C3	22.1	0.73	23.52	2.25
	T3	*51.74*	*1.36*	*38.48*	2.3
	G3	19.53	0.84	21.87	1.64
	G3 + C3	41.63	1.12	45.39	2.83
Codon usage	CAI-1^d^	0.76	0.01	0.71	0.04
	CAI-2^e^	0.65	0.02	0.59	0.04
	Ecai^f^	0.8	0.01	0.79	0.01

All the values are expressed as percentage

Significant differences are indicated in italics

*SD* standard deviation

^a,b,c^The numbers 1, 2 and 3 indicates the first, second and third position of a codon respectively

^d^Codon adaptation index calculated using CAIcal server

^e^Codon adaptation index calculated using GenScript Rare Codon Analysis Tool

^f^Expected codon adaptation index calculated using CAIcal server

### Secondary structures of the mRNAs and exposition of the translation initiation region

The secondary structures of the mRNA were predicted by the CentroidFold algorithm and are shown in Additional file 1: Figure S1. The mRNA secondary structures of the non-expressed genes were more stable than the ones of the expressed genes, with the free energies of − 11.57 ± 1.4 and − 8.14 ± 2.12 kcal/mol, respectively. The unpaired t test showed that the difference is statistically significant (P = 0.0277).

**Fig. 2:**
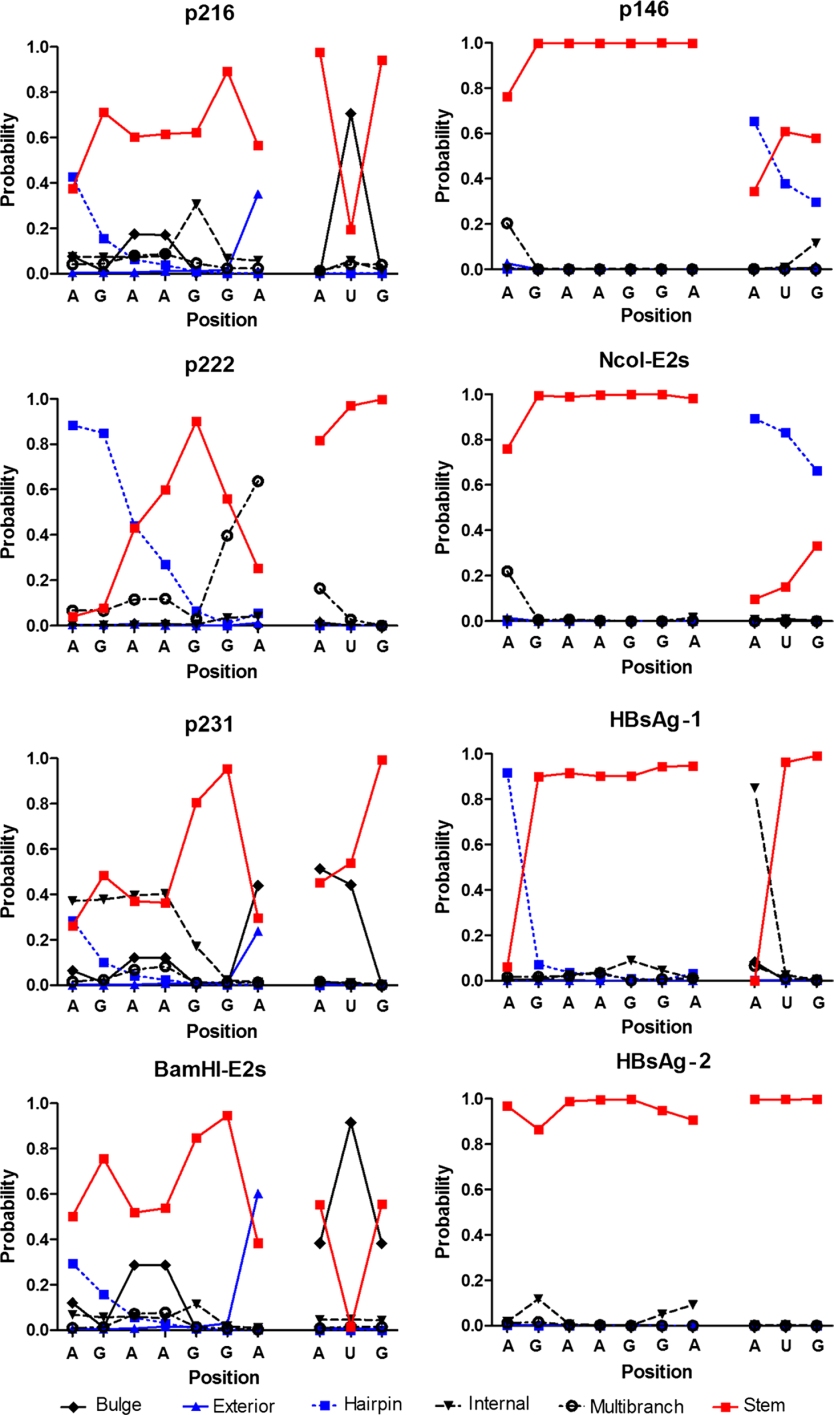
The probabilities of each RNA base position to be located within a specific secondary structural context as predicted by CapR algorithm. For the expressed genes (left), the nucleotides of the SD sequence are engaged in different secondary structural contexts. While in the non-expressed genes (right), the SD sequence nucleotides are engaged in a very stable stem-loop context

The RNA-binding proteins bind to their target RNA molecules by recognizing specific RNA sequences presented in specific structures. Therefore, we investigated the exposition of the SD sequence and the start codon using two different algorithms. First, we used the CapR algorithm to calculate the probabilities for each RNA base position to be within one of six different secondary structural contexts. The results showed that in the mRNA of the non-expressed genes, all of the base positions at the SD sequence were very likely to be in stems with a mean probability of 0.92 ± 0.12; while for the expressed genes, the probability of being in a stem context for the same base positions was lower (0.55 ± 0.2) (Fig. 2). The unpaired *t* test showed that the difference was significant with P = 0.0013. Further, in the expressed genes, the start codon sites included bulged nucleotides while in the non-expressed genes, the nucleotides at the start codon sites were mainly engaged in stems and internal loops for HBV genes (HBsAg-1 and HBsAg-2); and in stems and hairpin-loops for the HEV p146 and *Nco*I-E2s (Fig. 2).

**Fig. 3:**
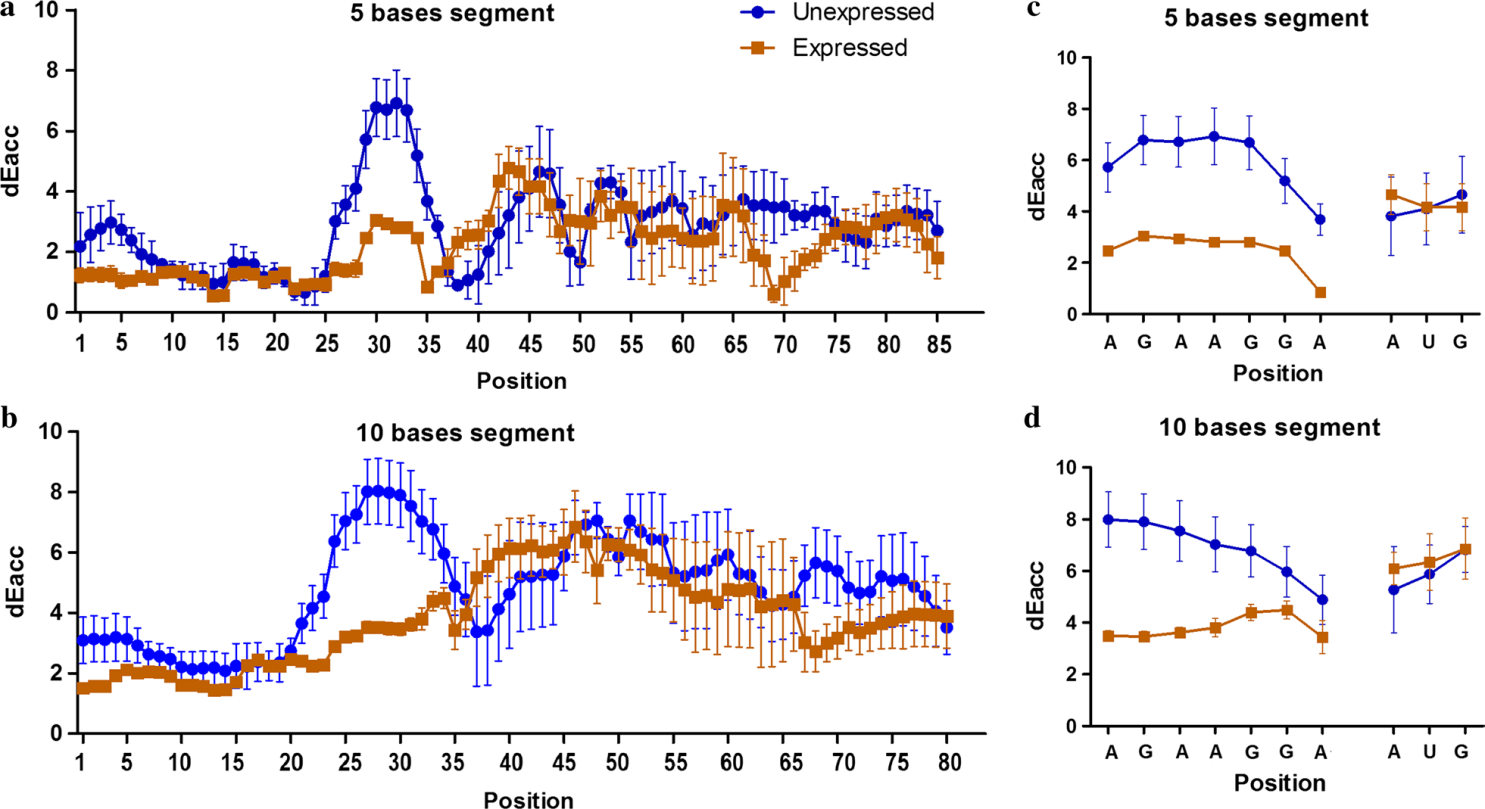
Accessibility of the start codon AUG and the SD sequence in the different mRNA secondary structures predicted using the Raccess algorithm. The results show the thermodynamic energy that is required to keep a given range of nucleotides being accessible. The more energy is needed the less the range is accessible. **a**, **b** The accessibility of each base in segments of 5 and 10 bases, respectively, all along the structure of the mRNA segment (− 43/AUG/+ 43). **c**, **d** The accessibility of SD sequence and initiator AUG bases in segments of 5 and 10 bases, respectively

Next, we used the Raccess algorithm to calculate the accessibility of the translation initiation region by calculating the thermodynamic energy that is required to keep a 5 bases or 10 bases segment accessible (see "Methods" section). The results in Fig. 3 showed the accessibility of each base position in the analyzed mRNA sequence when they are within a 5 bases and 10 bases segments (Fig. 3a, b respectively); and the accessibility of the SD sequence/start codon nucleotides in Fig. 3c, d respectively. These results indicated that the SD sequence in the mRNAs of the non-expressed genes needed higher thermodynamic energy to be accessible compared to the expressed genes (P = 0.0015) while no difference was noted concerning the start codon sites.

### Effects of single-point mutation on the overall stability of the mRNA secondary structure

**Fig. 4:**
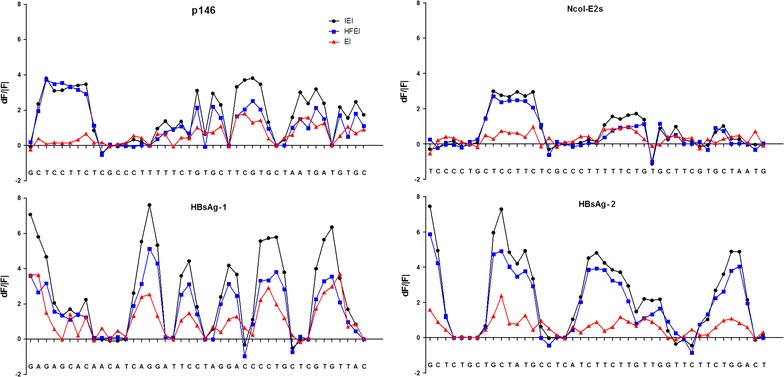
Increase of the overall energy of RNA secondary structures in response to single-point mutations. *IEI* internal energy increase, *HFEI* Helmholtz free energy increase, *EI* entropy increase

The results of RNA structure free energy fluctuations after the mutation of each single position are shown in Fig. 4. For the HEV non-expressed genes p146 and *Nco*I-E2s, the mutation of six positions located downstream of the start codons showed a significant increase in the overall free energy of the RNA secondary structures. Interestingly, these positions were composing the same codons (GCT, CCT and TCT): the first, second and third codons downstream the AUG in p146; and the third, fourth and fifth codons in *Nco*I-E2s. It is also worth noting that the mutation of the position upstream (only in *Nco*I-E2s) and downstream (in both p146 and *Nco*I-E2s) these three codons did not affect the overall stability of the RNA secondary structures. This indicated that these three codons participate actively in stabilizing the RNA secondary structures near the start codon and makes their mutation an interesting and reasonable approach to express p146 and *Nco*I-E2s proteins. Concerning the HBsAg-1 and HBsAg-2 genes, the results revealed that only the mutation of very few positions showed no energy change, indicating that most codons (more than 60%) were stabilizing the secondary structure of the mRNA.

### Analysis and expression of mutated p146 and *Nco*I-E2s genes

According to the Rchange results, the mutation of the codons 457 and 458 of p146 and *Nco*I-E2s would increase the overall free energy of the mRNA secondary structures (Fig. 4). The analysis of the free energy changes that would be induced by the different possible substitutions (Additional file 1: Figure S2) revealed that mutating the second base of the codon 457 (C with A) and the first and second positions of the codon 458 (CC with GA) would lead to the largest free energy increase in mRNA secondary structures for both p146 and *Nco*I-E2s.

**Fig. 5:**
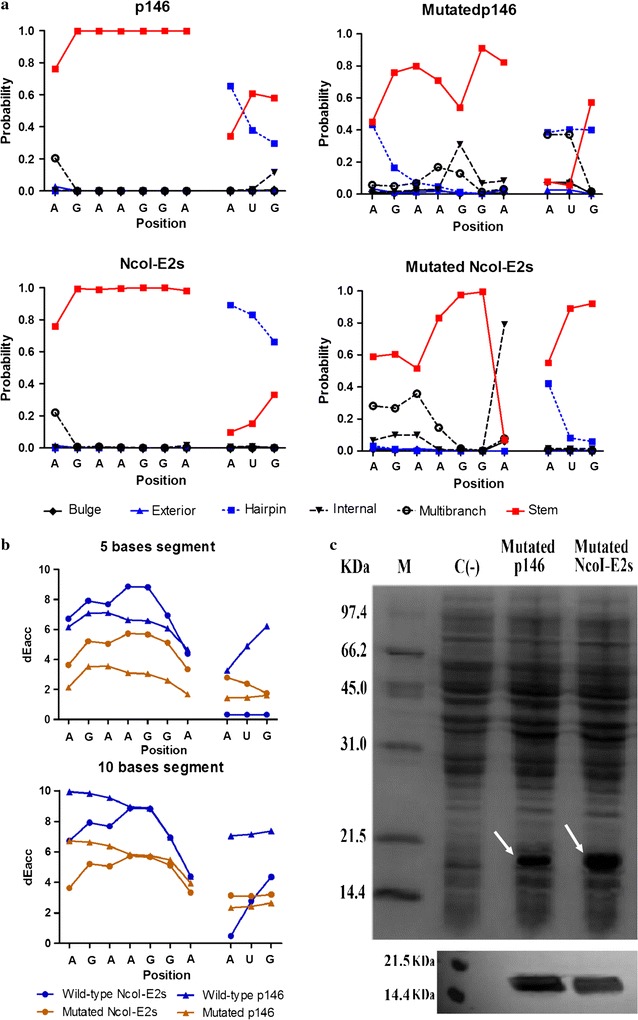
Analysis and expression of the mutated HEV p146 and *Nco*I-E2s. The same three mutations were introduced into p146 and *Nco*I-E2s: the second base of the codon 457 (C with A) and the first and second positions of the codon 458 (CC with GA). The predicted mRNA secondary structures near the start codons of the mutated genes shows that the start codon AUG and the SD sequence are exposed from the stable stem-loop structural context (**a**). The accessibility analysis (**b**) shows that the SD sequence in the mRNA of the mutated genes requires less thermodynamic energy to be accessible compared to the wild-type genes, indicating that the mutations allowed the exposition of the SD sequence. The mutated proteins were successfully over-expressed in *E. coli* (BL21) as shown by a 15% SDS-PAGE analysis (**c**, top) and were reactive against the anti-HEV 4C5 monoclonal antibody as shown by Western blot analysis (**c**, bottom)

The p146 and *Nco*I-E2s non-expressing genes were mutated, in the same three positions according to the above results: the second base of the codon 457 (C with A) and the first and second positions of the codon 458 (CC with GA). Then, the mRNA secondary structures near the start codon were analyzed as described previously and the results are shown in Fig. 5.

The overall free energies increased significantly from − 12.6 to − 2.9 kcal/mol in p146 and from − 10.04 to − 3.47 kcal/mol in *Nco*I-E2s (Additional file 1: Figure S3). Moreover, the CapR results indicated that the SD sequence and the start codon AUG were engaged in less stable secondary structure contexts in the mutated genes compared to the wild-types (Fig. 5a). Besides, these mutations decreased the required energy to be accessible for both the SD sequence and the initiator AUG, indicating that these regions became more exposed than in the wild-type genes (Fig. 5b). Taken together, these results indicated that the mutated p146 and *Nco*I-E2s were more likely to be expressed.

After introducing the three mutations, the mutated p146 and *Nco*I-E2s were successfully over expressed in *E. coli* (BL21) as shown in Fig. 5c top; and both proteins reacted against the HEV monoclonal antibody, indicating that the two mutated amino acids did not affect the exposure of the dominant neutralization epitopes (Fig. 5c bottom).

### Analysis and expression of mutated HBsAg-1 and HBsAg-2 genes

The Rchange results revealed that the mutation of the sixth codon GGA downstream the start codon in HBsAg-1 gene would induce the highest energy change in its mRNA secondary structure. Therefore, two substitutions have been introduced in the second and third position of this codon (GGA with GAT). Likewise, two codons have been mutated in the HBsAg-2 gene: the fourth and the fifth codons downstream the start codon (CTT and CTG respectively). The third positions of these codons have been substituted by T and G respectively. These two substitutions were chosen because they would increase the overall energy of the mRNA secondary structure more than all the other possible mutations, as computed by the Rchange algorithm (Additional file 1: Figure S2).

**Fig. 6:**
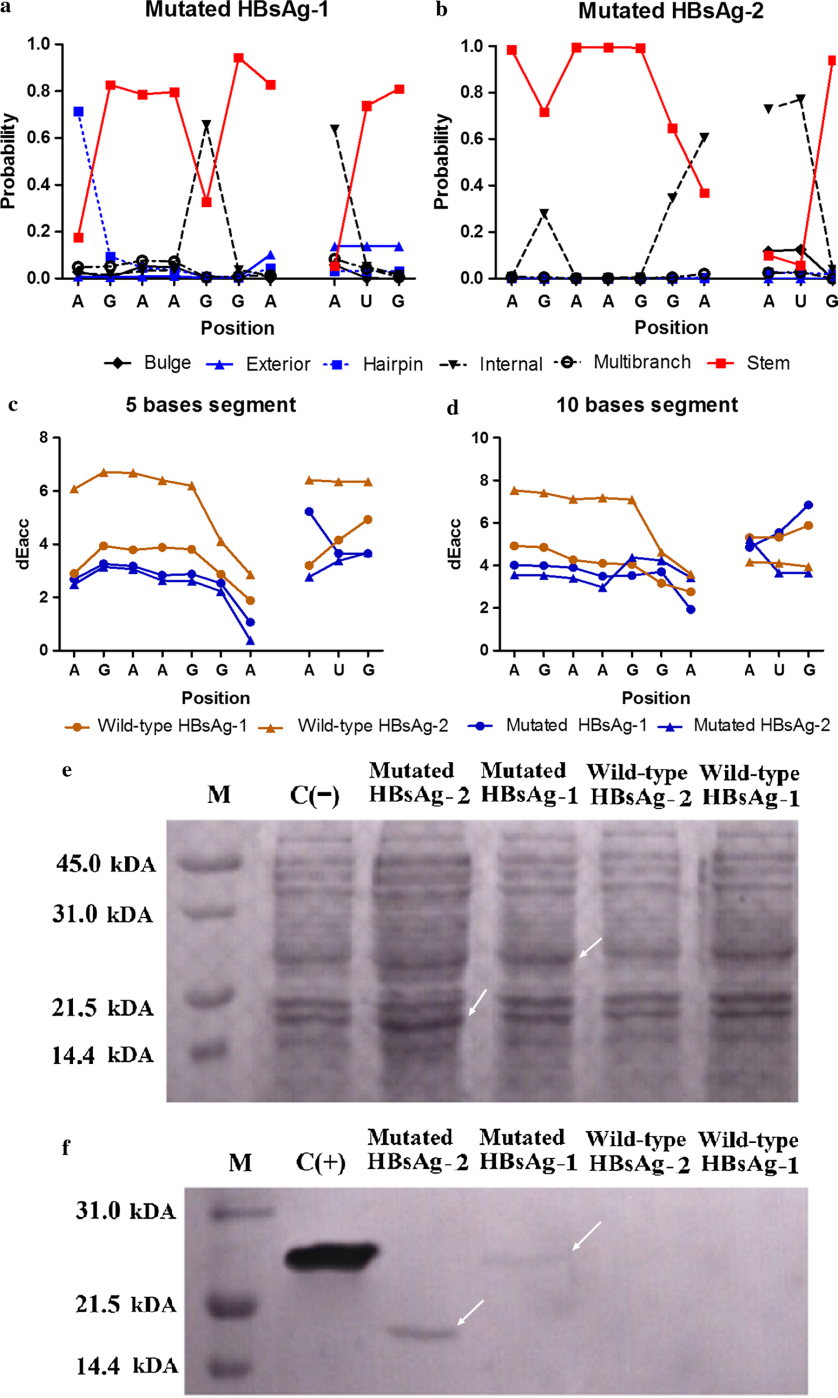
Analysis and expression of the mutated HBsAg-1 and HBsAg-2. The predicted mRNA secondary structures near the start codons of the mutated genes shows that the start codon AUG and the SD sequence are exposed from the stable stem-loop structural context (**a**, **b**). Furthermore, the accessibility analysis shows that the mutations allowed the exposition of the SD sequence in the mRNA of the mutated genes (**c**, **d**). The accessibility of the SD sequence in the mutated HBsAg-2 was significantly increased, while only a slight increase was noted in the mutated HBsAg-1. The expression of the mutated proteins was not very obvious in the SDS-PAGE analysis (**e**). However, the western blotting revealed weak reactive bands against the anti-HBsAg monoclonal antibody (**f**). This indicated that both mutated genes were weakly expressed

The mRNA secondary structures near the start codon of the mutated HBsAg-1 and HBsAg-2 were then analyzed as described previously and the results are shown in Fig. 6 and Additional file 1: Figure S3.

The overall free energies increased significantly from − 10.73 to 1.51 kcal/mol in HBsAg-1 and from − 12.89 to − 5 kcal/mol in HBsAg-2 (Additional file 1: Figure S3).

Moreover, the CapR calculations showed that the mutations introduced in HBsAg-1 and HBsAg-2 disrupted the formation of the stable stem-loops near the SD sequence and the start codon AUG (Fig. 6a, b). Besides, these mutations increased greatly the accessibility of the SD sequence and the initiator AUG in HBsAg-2 mRNA, but in HBsAg-1 only a slight increase in the accessibility has been noticed (Fig. 6c, d).

Next, the mutated HBsAg-1 and HBsAg-2 were successfully amplified, inserted into the pET28a(+) vector and the constructs were used to transform competent *E. coli* (BL21) cells. The expression of the HBsAg-1 and HBsAg-2 proteins was not very apparent in the SDS-PAGE of the whole expressing cells even after the optimization of the induction conditions (Fig. 6e). However, the Western blotting revealed weak bands at 26.5 and 17.1 kDa that correspond to the molecular weight of HBsAg-1 and HBsAg-2 proteins respectively (Fig. 6f). This indicated that both proteins were weakly expressed but fully reactive against the anti-HBsAg monoclonal antibody.

## Discussion

In this report, we investigated the expressivity of HEV and HBV recombinant proteins in *E. coli* when the respective genes are cloned under the control of the T7 promoter in the pET28a(+) vector. One of the strongest and most described expression systems in bacteria is based on the T7 RNA polymerase/promoter combination. The strength of the T7 RNA polymerase resides in its stringent specificity for its promoter and its selectivity in transcribing the DNA that has been linked to such a promoter [41]. Since the establishment of this system, it has been successfully used for the overexpression of heterologous proteins in *E. coli* [42, 43]. It has been previously shown that three different sequences located within the translation initiation region play an important role during translation, namely the Shine–Dalgarno sequence, the start codon and the region between them [44–47]. In this study, all the genes were inserted into the *Nco*I and *Xho*I restriction sites in order to keep the same spacer length between the SD sequence and start codon (8 nucleotides) for all the genes.

Three out of five HEV proteins were expressed p216, p222 and p231; while all HBV proteins were not. For this latter, the results were not unexpected, because for nearly half a century, the production of HBsAg in *E. coli* was unsuccessful in many attempts and of low efficiency in others [19–24]. Thus, the HBV vaccine is still produced in eukaryotic systems despite the complex procedure and relatively high cost compared to prokaryotic systems as discussed above. As for the reasons, they are not fully elucidated yet but some factors such as the codon bias, toxicity of the expressed protein for the host cells and the need of HBV 3′UTR have been reported to influence the expression of HBV genes [19, 48, 49]. Nonetheless, the stable mRNA secondary structure near the start codon, as reported here, seems to play a non-negligible effect in the expression of HBsAg. By introducing single-point mutations into the codons downstream the start codon we have increased the instability of the mRNA secondary structure of two HBV proteins and exposed the translation region, which in turn led to the expression of these HBsAg proteins. Although, the expression levels were too low, these data suggest the mRNA secondary structure should be taken into account during future attempts to produce HBsAg in *E. coli.*

The difference in codon usage between the different organisms can have a significant impact on heterologous protein production and the presence of rare codons can cause mistranslation events that prevent the expression of the target protein. It has been previously shown that the GC-rich codons tend to form more stable mRNAs structures and, therefore, the evolutionary forces tend to select AU-rich codons to ensure efficient translation [5, 7, 50]. This is in accordance with our findings in the present study, where the analysis of nucleotide composition of expressed and non-expressed genes revealed a significant difference in the A and U content at the second and third position respectively. The expressed genes had more A2/U3-rich codons compared to the non-expressed ones but no significant difference in the codon adaptation index. However, the analysis of amino acid composition and RSCU revealed that these discrepancies are due to the difference in protein composition between HEV and HBV. Nonetheless, the abundance of AU-rich codons in the HEV ORF2 coding sequences could explain their easy over-expression in *E. coli* [29–35].

The HEV p146 and *Nco*I-E2s were not expressed while the other HEV proteins were over-expressed even though they share mostly the same sequence. Therefore in the next step, we sought to determine the influence of the mRNAs secondary structures near the translation initiation region. The results revealed that indeed the mRNAs of the expressed genes had less stable secondary structures and this relative instability is, as reported by Gu et al. [51], a universal trend in both prokaryotes and eukaryotes; and it is a shared characteristic of functional genes in prokaryotes [9]. A stable mRNA structure can affect the accessibility of the specific RNA regions (SD sequence and the start codon AUG) recognizable by the ribosome [52] and it has been experimentally demonstrated that increased mRNA stability at the translation initiation region could prevent efficient translation initiation and hence decrease gene expression level [53, 54]. Here, the SD sequence in the mRNA of the non-expressed genes was engaged in a very stable stem-loop structure that made it inaccessible for the ribosome; on the contrary, in the expressed genes the SD sequence formed less stable bulge and hairpin structures and therefore was more accessible and this makes a plausible explanation why E2s was well expressed when inserted into the *Bam*HI–*Xho*I restriction sites but not in the *Nco*I–*Xho*I sites.

Further, we have introduced three point mutations to modify the 5′-end of the non-expressed p146 and *Nco*I-E2s according to the results obtained by analyzing single mutation effects on the overall stability of the mRNA secondary structure (Fig. 4). Interestingly, both secondary structures were stabilized by the same two codons (457 and 458) and their mutations allowed the increase of the overall free energy and the exposure of the SD sequence which led to the overexpression of both proteins. Similar results were previously obtained by Zhang et al. [55] when expressing the human IL-10 and IFN-α genes. They reported that introducing mutations into the 5′-end of the mRNA increased the overall free energy of the secondary structures and exposed the start codon AUG; and therefore permitted a significant increase in the expression efficiency of both genes. In the same stream, in synthetic biology, a highly reliable expression cassette design has been recently reported by Mutalik et al. [56] for the expression of heterologous genes in *E. coli*. This design was engineered to disrupt any secondary structure that does form near the SD sequence for the gene of interest by using another strong upstream ribosome binding site.

## Conclusions

Taken together, these results indicated that the mRNA stability near the translation initiation region and the AU-rich codons appear to play a non-negligible role for an efficient expression of the HEV ORF2 and HBV antigen surface genes in *E. coli*. The strong local mRNA secondary structure seemed to interfere with ribosome binding to the SD sequence but not with the start-codon recognition. However, given the small number of constructs analyzed in the present study, further investigation of large size libraries is needed.

Finally, we believe that the method used in here is very simple and would be very helpful for: (1) a first assessment of the expressivity of given heterologous gene in *E. coli* especially when aiming to produce and compare viral proteins of different lengths for vaccine or diagnostic antigens development; (2) design and optimization of the target genes; (3) the choice of the restriction sites in order to achieve high expression levels.

## Additional file

**Additional file 1.** Additional tables and figures.
