# Supplementary material for: Effects of mRNA secondary structure on the expression of HEV ORF2 proteins in Escherichia coli
Source: Microb Cell Fact. 2017 Nov 14;16:200. doi: 10.1186/s12934-017-0812-8 (PMC5686824; doi:10.1186/s12934-017-0812-8)
Supplement: Supplementary file 1 — Additional file 1. Additional tables and figures. [file 12934_2017_812_MOESM1_ESM.zip › Supplementary tables.pdf]

Legends of the supplementary figures

Figure S1. The mRNA secondary structure near the start codon AUG predicted using Centroidfold algorithm. The mRNA secondary structures of the expressed genes are presented on the left and those of the unexpressed genes on the right. Green arrows indicate the first position of the start codon AUG (nt 44-46); black arrows indicate the first position of the SD sequence (nt 29-35). The overall free energy of each secondary structure (kcal/mol) is shown in between brackets.

Figure S2. Screen-shots of the Rchange output files of p146, NcoI-E2s, HBsAg-1 and HBsAg-2. The output data are presented as follows: Sequence positions of mutations (mut_pos1, mut_pos2) are in 0-based indexing convention. "mutation_data" is semicolon (';') delimited entries representing different mutations to the original base(s). For each mutation, there are 4 fields delimited by comma ',' representing mutated base(s), entropy change, internal energy change, Helmholtz free energy change.

The data of the mutated position are highlighted in yellow. For, each position, the highest free energy increase induced by a single mutation is highlighted in green while the energy increases induced by the other possible substitutions are highlighted in red.

Figure S3. The predicted mRNA secondary structures near the start codons of the mutated p146, NcoI-E2s, HBsAg-1 and HBsAg-2 genes shows that the start codon AUG (red arrows) and the SD sequence (black arrows) are exposed from the stable stem-loop structural context.

Table S1. Amino acid composition of HEV and HBV target proteins

AA	HEV target proteins		HBV target proteins	
	Avg.	S.D.	Avg.	S.D.
Phe	5.00	1.41	13.67	3.51
Leu	15.88	2.75	29.33	6.03
Ile	6.00	1.41	11.00	3.00
Val	15.38	1.92	9.33	0.58
Ser	18.88	2.42	20.33	5.51
Pro	11.00	2.62	20.00	4.58
Thr	20.25	1.98	15.33	4.62
Ala	16.13	3.04	5.33	1.15
Tyr	10.38	1.30	4.00	1.00
His	3.50	2.33	0.67	0.58
Gln	7.63	2.56	5.67	2.31
Asn	7.50	0.53	3.67	1.53
Lys	4.88	1.36	2.00	0.00
Asp	10.13	4.19	2.67	0.58
Glu	5.25	2.43	1.67	0.58
Cys	3.00	1.41	12.33	2.08
Arg	8.25	2.43	4.67	2.31
Gly	12.50	2.83	11.67	4.04
Met	2.75	1.39	5.67	0.58
Trp	3.00	0.00	11.67	1.53

HEV target proteins: p166, p179, p216, p222, p231, p146 and E2s

HBV target proteins: HBsAg-1, HBsAg-2 and HBsAg-3

AA: amino acids

Avg.: average; S.D.: standard deviation

Table S2. Relative synonymous codon usage (RSCU) in HEV and HBV target genes

AA[a]	CODONS	HEV genes		HBV genes	
		Avg.[b]	S.D.[c]	Avg.	S.D.
**Phe**	**TTT**	**80.0**	5.5	39.0	3.0
	**TTC**	20.0	5.5	**61.0**	3.0
**Leu**	**TTA**	0.0	0.0	10.3	3.3
	**TTG**	15.8	4.7	17.0	0.3
	**CTT**	**42.5**	2.2	16.5	4.5
	**CTC**	14.8	1.3	15.3	5.8
	**CTA**	6.0	0.8	**22.7**	0.8
	**CTG**	21.0	4.0	18.2	1.7
**Ile**	**ATT**	37.3	4.3	28.0	9.7
	**ATC**	**61.0**	4.7	**42.0**	4.0
	**ATA**	2.0	4.3	30.0	5.7
**Val**	**GTT**	**48.3**	3.5	**43.0**	2.5
	**GTC**	25.8	3.0	21.5	1.3
	**GTA**	6.3	0.5	17.8	6.0
	**GTG**	20.0	4.3	17.8	6.0
**Ser**	**TCT**	**61.0**	7.8	16.2	1.5
	**TCC**	17.8	2.2	21.3	0.5
	**TCA**	6.8	2.7	**26.5**	1.8
	**TCG**	5.2	0.5	10.8	3.2
	**AGT**	5.2	0.5	22.2	5.7
	**AGC**	4.2	10.2	2.8	2.5
**Pro**	**CCT**	**67.3**	2.0	28.3	1.3
	**CCC**	13.3	6.5	30.3	2.5
	**CCA**	18.0	3.0	**41.5**	1.5
	**CCG**	1.8	4.5	0.0	0.0
**Thr**	**ACT**	**80.3**	2.3	24.8	4.5
	**ACC**	10.3	2.8	**28.5**	1.3
	**ACA**	4.8	0.5	**28.8**	7.8
	**ACG**	4.8	0.5	17.8	2.0

AA	CODONS	HEV genes		HBV genes	
		Avg.	S.D.	Avg.	S.D.
**Ala**	**GCT**	**55.5**	4.3	**38.8**	9.8
	**GCC**	25.3	2.0	19.5	4.8
	**GCA**	16.8	2.8	30.5	4.8
	**GCG**	2.5	2.8	11.3	9.8
**Tyr**	**TAT**	**64.5**	2.5	44.0	21.0
	**TAC**	35.5	2.5	**56.0**	21.0
**His**	**CAT**	**98.0**	5.0	0.0	0.0
	**CAC**	2.0	5.0	**66.5**	57.5
**Gln**	**CAA**	16.5	9.0	**51.0**	13.5
	**CAG**	**84.0**	9.0	49.0	13.5
**Asn**	**AAT**	**87.0**	1.0	45.0	39.5
	**AAC**	13.0	1.0	**55.0**	39.5
**Lys**	**AAA**	30.0	10.0	**50.0**	0.0
	**AAG**	**70.0**	10.0	**50.0**	0.0
**Asp**	**GAT**	**70.0**	8.0	0.0	0.0
	**GAC**	30.0	8.0	**100.0**	0.0
**Glu**	**GAA**	0.0	0.0	0.0	0.0
	**GAG**	**100.0**	0.0	**100.0**	0.0
**Cys**	**TGT**	42.5	10.0	**58.5**	7.5
	**TGC**	**57.5**	10.0	41.5	7.5
**Arg**	**CGT**	38.3	7.2	**38.8**	9.7
	**CGC**	**43.8**	6.2	11.2	9.7
	**CGA**	3.0	4.8	0.0	0.0
	**CGG**	14.7	3.0	11.2	9.7
	**AGA**	0.0	0.0	**38.8**	9.7
	**AGG**	0.0	0.0	0.0	0.0
**Gly**	**GGT**	30.0	1.8	9.5	4.0
	**GGC**	**41.3**	1.3	9.5	8.3
	**GGA**	2.0	4.8	**52.5**	4.3
	**GGG**	27.0	4.8	28.5	0.0

The values are expressed as percentage

The preferentially used codons for each amino acid are indicated in bold.

[a] amino acids; [b] Average; [c] standard deviation.

Table S3.

Genes	Sequences of the -43/ATG/+43 mRNA segments
**NcoI-E2s**	CCUCUAGAAAUAAUUUUGUUUAACUUUAAGAAGGAGAUAUACCAUGUCCCCUGCUCCUUCUCGCCCUUUUUCUGUGCUUCGUGCUAAUG
**Mutated NcoI-E2s**	CCUCUAGAAAUAAUUUUGUUUAACUUUAAGAAGGAGAUAUACCAUGUCCCCUG**A**U**GA**UUCUCGCCCUUUUUCUGUGCUUCGUGCUAAUG
**p146**	CCUCUAGAAAUAAUUUUGUUUAACUUUAAGAAGGAGAUAUACCAUGGCUCCUUCUCGCCCUUUUUCUGUGCUUCGUGCUAAUGAUGUGC
**Mutated p146**	CCUCUAGAAAUAAUUUUGUUUAACUUUAAGAAGGAGAUAUACCAUGG**A**U**GA**UUCUCGCCCUUUUUCUGUGCUUCGUGCUAAUGAUGUGC
**HBsAg-1**	CCUCUAGAAAUAAUUUUGUUUAACUUUAAGAAGGAGAUAUACCAUGGAGAGCACAACAUCAGGAUUCCUAGGACCCCUGCUCGUGUUAC
**Mutated HBsAg-1**	CCUCUAGAAAUAAUUUUGUUUAACUUUAAGAAGGAGAUAUACCAUGGAGAGCACAACAUCAG**AT**UUCCUAGGACCCCUGCUCGUGUUAC
**HBsAg-2**	CCUCUAGAAAUAAUUUUGUUUAACUUUAAGAAGGAGAUAUACCAUGGCUCUGCUGCUAUGCCUCAUCUUCUUGUUGGUUCUUCUGGACU
**Mutated HBsAg-2**	CCTCTAGAAATAATTTTGTTTAACTTTAAGAAGGAGATATACCATGGCTCTGCT**T**CT**G**TGCCTCATCTTCTTGTTGGTTCTTCTGGACT

The SD sequences are highlighted in green

The start codons are highlighted in yellow

The substitutions in the mutated genes are highlighted in cyan
