# Supplementary figures and images for: Effects of mRNA secondary structure on the expression of HEV ORF2 proteins in Escherichia coli
Source: Microb Cell Fact. 2017 Nov 14;16:200. doi: 10.1186/s12934-017-0812-8 (PMC5686824; doi:10.1186/s12934-017-0812-8)

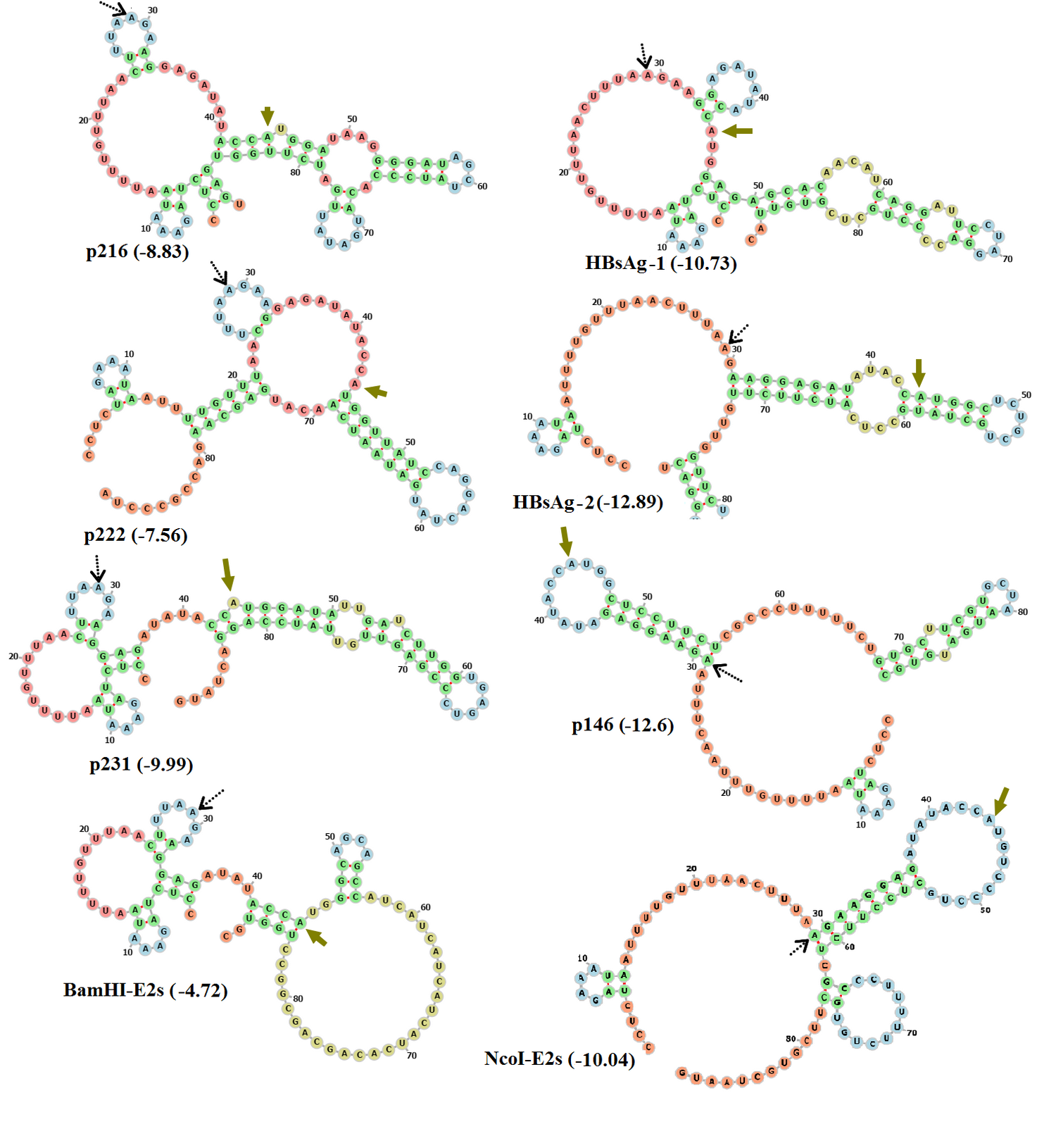

Supplement: Supplementary file 1 — Additional file 1. Additional tables and figures. [file 12934_2017_812_MOESM1_ESM.zip › Figure S1 RNA structures.png]

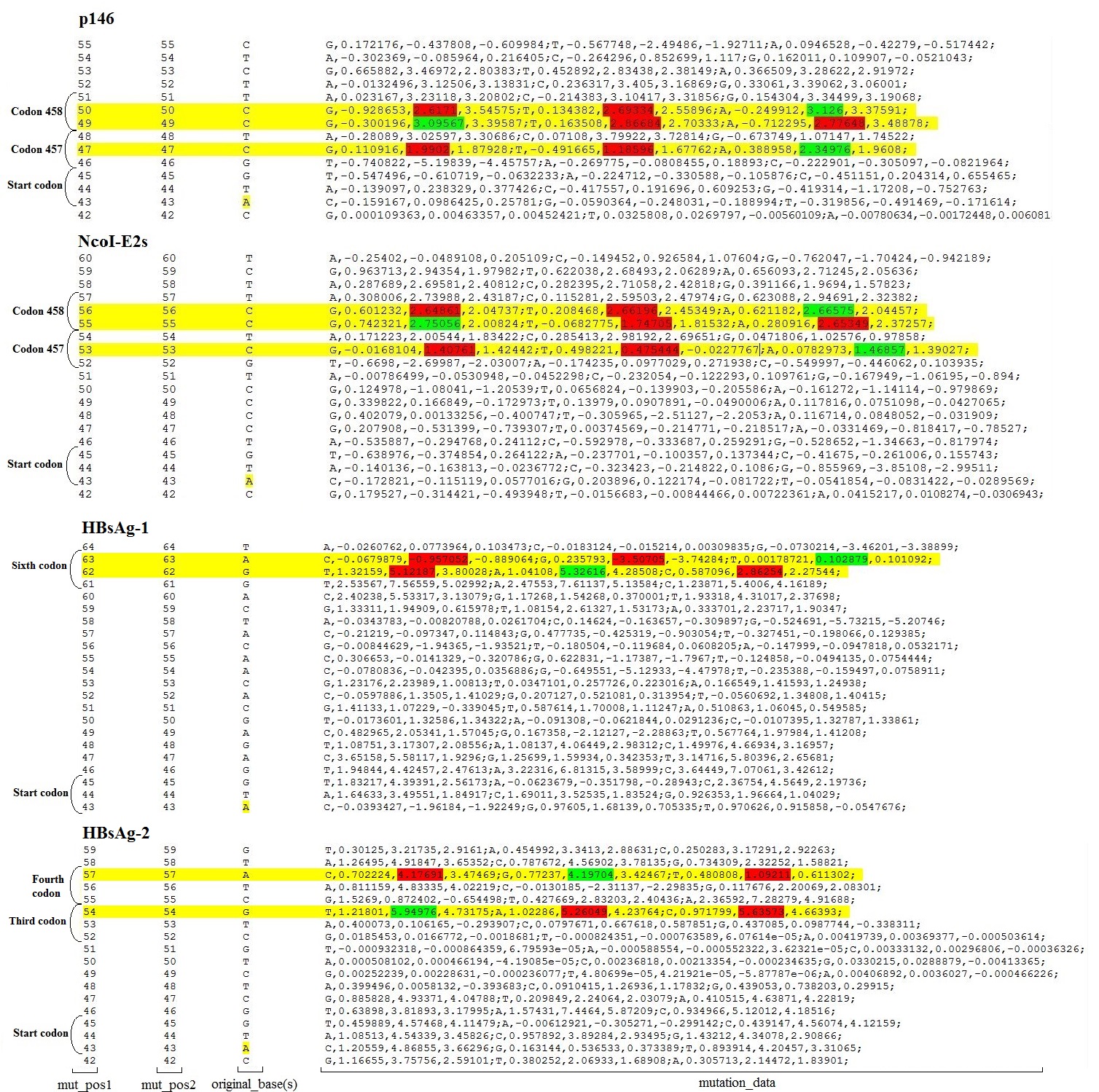

Supplement: Supplementary file 1 — Additional file 1. Additional tables and figures. [file 12934_2017_812_MOESM1_ESM.zip › Figure S2.JPG]

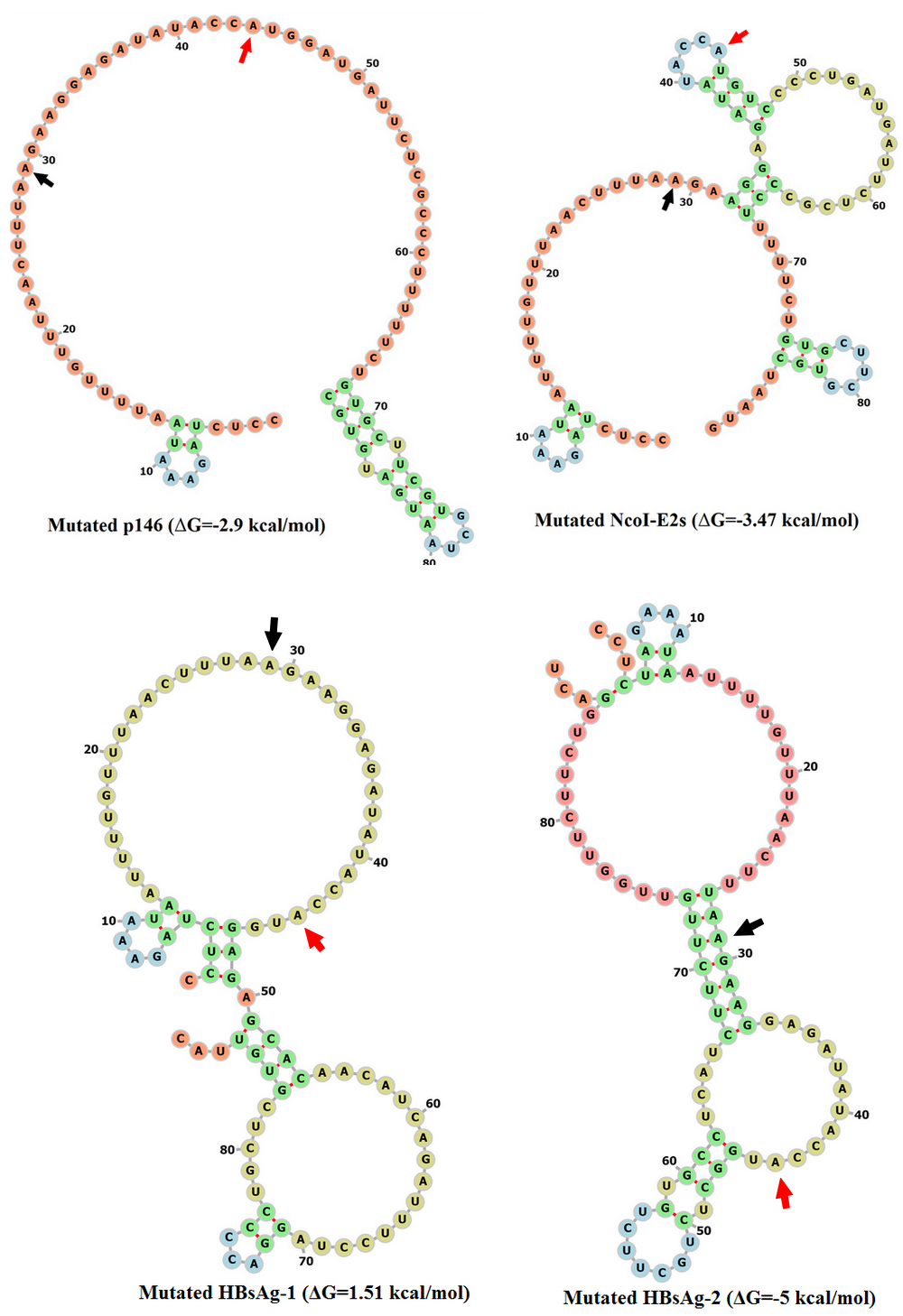

Supplement: Supplementary file 1 — Additional file 1. Additional tables and figures. [file 12934_2017_812_MOESM1_ESM.zip › Figure S3.tif]
